# Application of allograft and absorbable screws in the reconstruction of a massive bone defect following resection of giant osteochondroma: A retrospective study

**DOI:** 10.3389/fsurg.2022.938750

**Published:** 2022-09-22

**Authors:** Zhihao Ma, Qiang Yang, Xinyu Liu, Zhenfeng Li

**Affiliations:** Department of Orthopedics, Qilu Hospital of Shandong University, Jinan, China

**Keywords:** osteochondroma, massive bone defect, allograft, absorbable screw, reconstruction

## Abstract

**Background:**

This study aims to introduce a reconstruction method of applying allografts and absorbable screws to repair large bone defects caused by the resection of giant osteochondroma.

**Methods:**

A retrospective study of a series of patients who underwent the resection of giant osteochondroma reconstructed by allografts and absorbable screws was conducted from February 2020 to September 2021. Their demographic data, location site, area of bone defect, and pertinent operative details were recorded. The reconstruction modality of allografts was elaborated on. In the follow-up, radiographic images were utilized to determine bone union, and the Musculoskeletal Tumor Society score was used to evaluate postoperative limb function.

**Results:**

A total of seven patients were included, including three males and four females with an average age of 16.6 ± 6.5 years. Among them, three cases of tumors occurred in the humerus and four cases occurred in the femur. The average follow-up time was 11.3 ± 3.0 months. The average area of bone defect was 25.9 ± 8.3 cm^2^. No complications such as infection, nonunion, and allograft bone fracture were found during the follow-up period. Six months after the operation, the average Musculoskeletal Tumor Society score was 26.4 ± 1.6, with acceptable postoperative function.

**Conclusions:**

The cooperative application of absorbable screw fixation and allografts including mixed cortical bone and cancellous bone, which yielded satisfactory functional outcomes and acceptable postoperative complications, is an effective reconstruction method for a massive bone defect after the resection of giant osteochondroma.

## Introduction

Osteochondroma is the most common benign bone tumor. Most osteochondromas are asymptomatic. The main symptoms are mechanical compression, fracture, bursitis, or malignant transformation of adjacent structures (1–4). At present, the main surgical treatment of osteochondroma is tumor resection, which aims to excise the tumor beyond a safe margin, maintain biomechanical support, and achieve satisfactory postoperative functional outcomes. Complete resection of the exostosis, cartilage cap, and perichondrium from the base of normal bone is the recommended intervention. If the resection region of the tumor is inadequate or residuals of cartilage and perichondrium remain, there is a high risk of recurrence (5–8). A wide surgical margin aims to reduce the risk of tumor recurrence. However, for some large osteochondromas, marginal resection of the tumor without reconstruction will no doubt leave a massive bone defect inevitably. This massive bone defect may lead to unsatisfactory postoperative functional outcomes.

According to the previous studies on the biomechanical analysis of the defect in long bone, if the length of the defect exceeds a certain degree, it will affect the shear strength and antibending load of the remaining shaft after tumor resection (9–11). Therefore, it is necessary to actively carry out reconstruction surgery following the resection of the tumor to restore its biomechanical stability. We proposed a surgical technique of using allograft cortical bone and cancellous bone combined with absorbable screw fixation to reconstruct the huge bone defect after osteochondroma resection. The usage of allografts can provide mechanical support for bone defects. At the same time, cancellous bone was used to fill the gap left after the bone graft, which can increase the bone contact area between the allograft and host bone. The enlarged contact surface is capable of accelerating the process of creeping substitution between the allograft and host bone and can achieve ideal osteoinductivity to shorten the time of bone graft fusion. In addition, absorbable screws were used to ensure a temporary fixation for the brittler texture of allograft. Absorbable screws reduce the use of metal materials, which can save the patients from the pain of removing the internal fixation again in the future. In addition, absorbable screws had the advantage of reducing the opacity effect of metals in radiographs and reducing metal artifacts in computed tomography and magnetic resonance imaging.

The purpose of this study is to evaluate the feasibility of our reconstruction technique for a bone defect.

## Materials and methods

### Demographics

Based on the hospital data, we searched the medical records of all patients who underwent osteochondroma research from February 2020 to September 2021. The exclusion criteria were that the transverse diameter of osteochondromas did not approach 25% of the width of the bone. A total of seven patients (three males and four females) who received allografts were included in this study. Patients were followed for a minimum of 7 months (mean, 11.3 months; range, 7–14 months). Pertinent operative details such as surgery duration, estimated blood loss, and complications were also recorded (Table 1). This retrospective study was approved by our institutional review committee. All participants agreed with the data and publication of the manuscript.

**Table 1:** Osteochondroma patients’ demographics (*n* = 7).

Demographic	Value
Age (years)	16.6 ± 6.5
Sex, male:female	3:4
Location	
Humerus	3
Femur	4
Area of bone defect (cm^2^)	25.9 ± 8.3
Surgery duration (min)	173.6 ± 65.2
Intraoperative blood loss (ml)	181.4 ± 66.4
Follow-up time (months)	11.3 ± 3.0
Musculoskeletal Tumor Society score at 6 months after the operation	26.4 ± 1.6

Values are presented as mean ± standard deviation or number.

### Surgical technique

**Figure 1:**
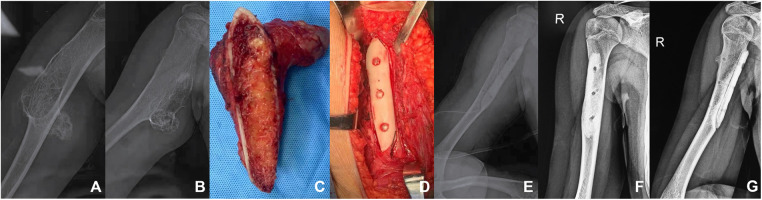
A patient with humerus osteochondroma. (**A,B**) The preoperative plain radiographs of the patient showed a huge osteochondroma at the proximal end of the right humerus, with a size of 12 cm * 5 cm * 5 cm. (**C**) Osteotomy was performed from the base of the tumor. (**D**) The allograft was implanted in the bone cortical defect and fixed with three absorbable screws. (**E**) Immediate postoperative radiographs. (**E,F**) Six months after the operation, the plain radiographs of the right humerus showed that the effect of bone graft fusion was desirable.

After the success of general anesthesia, the patients were laid in a supine position, and the operation area was routinely disinfected and wrapped. The incision was selected according to the tumor's location, the skin, subcutaneous tissue, and deep fascia were cut in turn, the muscle and periosteum were stripped off, the tumor was exposed, and the tumor tissue was separated from the surrounding normal tissue. During the operation, piezosurgery was used to cut the edge of the tumor, dished to remove the tumor, and sent to routine pathological examination. After the allograft cortical bone plate and cancellous bone were properly repaired in accordance with the shape of the bone defect, the allogeneic bone plate and cancellous bone were implanted into the bone cortical defect. Then, three absorbable screws were fixed for the sake of temporary stability. Intraoperative radiographs were reviewed to assess the effect of bone graft. Antibiotics were used once before the operation (Figure 1).

### Clinical follow-up

**Figure 2:**
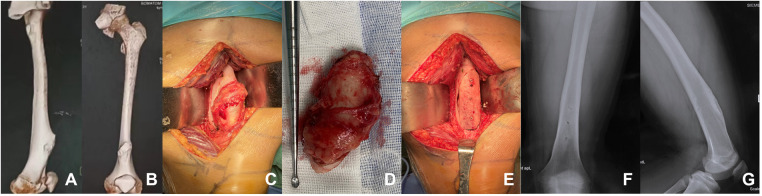
A patient with femoral osteochondroma. (**A,B**) The preoperative three-dimensional reconstruction of the patient showed a huge osteochondroma at the left femoral. (**C–E**) Osteochondroma resection was performed from the base of the tumor. The allograft was implanted in the bone cortical defect and fixed with three absorbable screws. (**F,G**) One year after the operation, the radiographs of the left femur showed that the effect of bone graft fusion was satisfactory.

Plain radiography examination was performed every 3 months after the operation to evaluate the implantation and fusion of allogeneic bone, supplemented by the weight-bearing capacity of the target limb and the local clinical symptoms such as tenderness and vertical percussed pain at the surgical site (Figure 2). Patients were assessed by the Musculoskeletal Tumor Society score 6 months after the operation. In the process of follow-up, we attached great importance to the movement of limbs of patients and their postoperative rehabilitation.

## Results

The mean age of the patients was 16.6 years, ranging from 13 to 31 years. In this study, three patients had tumors in the humerus and four had tumors in the femur. The mean area of bone defect was 25.9 ± 8.3 cm^2^. The average time of surgery was 173.6 ± 65.2 min. The estimated intraoperative blood loss was 181.4 ± 66.4 ml. The average follow-up was 11.3 ± 3.0 months, ranging from a minimum of 7 months to a maximum of 14 months. None of the patients was lost to follow-up, and none of them had complications such as infection, recurrence, allograft fracture, delayed union, and nonunion for the time being. Based on the Musculoskeletal Tumor Society functional evaluation, the mean score 6 months after the operation was 26.4 ± 1.6. Plain radiographs showed signs of bone union three months after the operation, and only a few patients still had tenderness and vertical percussed pain at the surgical site. At the second follow-up, all patients had no restrictions on daily life. However, some strenuous exercises were still limited to patients to prevent fractures at the operative site.

## Discussion

At present, the recommended surgical intervention for osteochondroma is marginal resection. However, a large bone defect will be left after complete resection of the giant osteochondroma, although the definition of the size of a massive bone defect has not been well determined (12). Amanatullah et al. suggested in the biomechanical study of long bone defects that the area of the cortical defect had a negative correlation with the hardness of defective bone (11). Moreover, the average torsional stiffness has a strong linear correlation with the size of the cortical defect, so the remaining bone after resection of the bone defect is of great significance to its stability. These biomechanical analyses predicted a severe loss of torsional integrity when the cortical defect approaches 50% of the width of the femur. Simply resecting the osteochondroma without reconstruction would inevitably leave a large bone defect. The remaining cortical bone cannot obtain sufficient biomechanical support, and fracture may occur. Therefore, it is necessary to actively carry out reconstruction surgery after the resection of osteochondroma to restore the biomechanical stability of defective bone (9–11).

A reconstruction method that applicating allograft and absorbable screws to repair large bone defects caused by the resection of giant osteochondroma is introduced in this study. The transverse diameter of cortical defect, which approached 25% of the width of the bone, or the longitudinal diameter of cortical defect, which exceeded the width of the bone, is more suitable for this method. Allograft is used to obtain the strength and mechanical stability of the reconstructed bone in our surgical procedure. In the process of bone union, the contact area of allograft and host bone needs enough matching and absolute long-term stability to complete the slow process of creeping substitution. Therefore, the allograft needs long-term protection to share the stress beyond its supporting capacity. At the same time, allogenic cancellous bone is applied to fill in the medulla to preserve the bone mass after the defect as much as possible. The application of cancellous bone could fill the gap left after the bone graft so that it can increase the bone contact area between the allograft and the host bone. The enlarged contact surface is capable of accelerating the process of creeping substitution between the allograft and host bone, which could shorten the time of bone graft fusion. The usage of cancellous bone can also induce ideal osteoinduction and osteoconduction to achieve satisfactory bone union. Absorbable screws are used to provide short-term support for allografts before bone fusion.

In our study, three patients had tumors in the humerus and four had tumors in the femur. Due to the femur and tibia being the main weight-bearing bones of the human body, the stress load after reconstruction is greater. Thus, for some patients with larger femoral defects, the allograft is fixed with metal plates and screws to strengthen the mechanical stability of the affected limb. Regarding the enhancement of the stability of allograft and reduction of the risk of fracture, Gupta et al. reported that allograft augmented with intramedullary cement and plate fixation is a reliable solution (13, 14). Being the nonweight-bearing bone, the humerus has a lower bearing requirement than the femur or tibia. Therefore, we added absorbable screws for fixation instead of traditional metallic screws, and the implantation of metal plates was not necessary. Compared with the traditional metal plate and screw fixation, our technique can reduce the use of metal materials, which can save the patients from the pain of removing the internal fixation again in the future. Although the metal plate and screw internal fixation are more stable, the use of absorbable screws can also reach a favorable short-term fixation. In addition, absorbable screws have the advantages of reducing the opacity effect of metals in plain radiographs and metal artifacts in computed tomography and magnetic resonance imaging, so we are able to make an early assessment of the fusion process of cortical cancellous bone and local recurrence (15, 16). Another benefit is that postoperative pain associated with elastic modulus mismatch may be reduced. Compared with metal plates and screws, cortical allogeneic bone scaffolds can better reconstruct the biomechanical elastic modulus of bone. In some published studies, this mismatch was considered to be one of the considerable causes of postoperative pain and eventual implant failure (17–19). The price of absorbable screws is also higher.

Because the texture of allograft is brittler than that of normal bone, it is difficult for other tools to grind it into a suitable shape. As a consequence, piezosurgery was used in the process of operation to cut the interface between host bone and allograft directly into the bevel to increase the contact area of biological bone graft and make it more matched, which could shorten the healing time and enable patients to recover and exercise early. At the same time, we ground the growth axis of the bone defect into an oval in the same direction as the length and diameter of the bone according to precise match orientation to better adapt to the biomechanics of the reconstructed bone.

Although the allograft has been used as a biological bone preservation technique, several studies have reported that there remain several potential problems such as allograft fracture (20–22), infection (20–24, 25), delayed union, and nonunion (5, 25–27). Aponte-Tinao et al. reported 6 infections and 4 fractures in 80 osteoarticular distal femur allografts (20). Aponte-Tinao et al. reported in another study that the incidence of infection of the allograft was 9% (23). Sorger et al. found in their study that 17.7% of structural allografts fractured at a mean of 3.2 years after transplantation (22). A retrospective study reported that 2 of 25 patients had postoperative infection (25). In our case, none of the patients had these complications above for the time being. Although it is generally believed that the incidence of allografts is high, we found that the incidence of postoperative infection, fracture, and delayed bone union is low in our study. One possible reason is that our technique can shorten the operation time, reduce intraoperative bleeding, and reduce the incidence of short-term and long-term postoperative complications. Another reason may be that our sample size is so small that the accuracy of the results may be compromised. As a result, patients were able to carry out simple rehabilitation training early. The passive functional exercise was necessary and could be carried out as soon as possible after the operation, which can prevent muscle atrophy and anchylosis. However, the active movement of the affected limb should be restricted in the short term to avoid fractures caused by excessive load or rotational violence.

There are several limitations, although our method of operation was very effective. First, our study was a retrospective study, which lacked a direct comparison with other treatment techniques, especially plate and screw fixation, so further case–control studies are needed to investigate whether we have the advantage. At the same time, we currently had an average follow-up period of 11.3 ± 3.0 months, which was still shorter than that reported in relevant studies. And the population was relatively small as a result of the rarity of the procedure, so late follow-up is needed to evaluate whether there would be new long-term complications. In addition, our research can be combined with the prevalent 3D printing technology so that we can better plan the bone defect and its reconstruction, including the development of personalized guide plates and vascularized stent (28–30). However, there also exist some shortcomings, such as high cost and a long learning cycle. In addition, the long-term outcomes and the remedy after the failure of 3D printing technology have not been certified clearly (31).

## Conclusions

In the reconstruction of the massive bone defect after the operation of osteochondroma of a long bone, the use of allografts combined with absorbable screw fixation is an effective method of reconstruction, which achieves favorable surgical outcomes. It is safe in terms of the risk of infection and allograft fracture. Considering that it can reduce the use of metal plates and screws, it also has some advantages in reducing the metal artifacts of computed tomography or magnetic resonance imaging. In addition, because the stiffness of the allograft is closer to the bone than metal, the limb pain associated with the difference in elastic modulus mismatch can be reduced in the meantime.

## Data Availability

The raw data supporting the conclusions of this article will be made available by the authors, without undue reservation.
